# A review of the John F. Kennedy Medical Center's response to the COVID-19 pandemic in Liberia

**DOI:** 10.3389/fpubh.2023.1258938

**Published:** 2024-01-09

**Authors:** Ian Wachekwa, Sia Wata Camanor, Tete Kpoeh-Thomas, Facia Glaydor, Yassah Moracious Barclay-Korboi, J. Soka Moses, Joyce Weade Bartekwa-Gwaikolo

**Affiliations:** ^1^Department of Internal Medicine, John F. Kennedy Medical Center, Monrovia, Liberia; ^2^Office of the Chief Medical Officer, John F. Kennedy Medical Center, Monrovia, Liberia; ^3^Infection Prevention and Control Unit, John F. Kennedy Medical Center, Monrovia, Liberia; ^4^Epi-Surveillance, John F. Kennedy Medical Center, Monrovia, Liberia; ^5^Partnership for Research on Vaccines and Infectious Diseases in Liberia, Monrovia, Liberia

**Keywords:** COVID-19, pandemic, John F. Kennedy Hospital, health system strengthening, Liberia

## Abstract

**Objective:**

Over the past decades, the world has experienced a series of emerging and re-emerging infectious disease pandemics with dire consequences for economies and healthcare delivery. Hospitals are expected to have the ability to detect and respond appropriately to epidemics with minimal disruptions to routine services. We sought to review the John F. Kennedy Medical Center's readiness to respond to the COVID-19 pandemic.

**Methods:**

We used the pretest-posttest design in June 2021 and May 2023 to assess the hospital's improvements in its COVID-19 readiness capacity by collecting data on the hospital's characteristics and using the WHO COVID-19 Rapid hospital readiness checklist. We scored each readiness indicator according to the WHO criteria and the hospital's overall readiness score, performed the chi-square test for the change in readiness (change, 95% CI, *p*-value) between 2021 and 2023, and classified the center's readiness (poor: < 50%, fair: 50–79%, or satisfactory: ≥80%). The overall hospital readiness for COVID-19 response was poor in 2021 (mean score = 49%, 95% CI: 39–57%) and fair in 2023 (mean score = 69%, 95% CI: 56–81%). The mean change in hospital readiness was 20% (95% CI: 5.7–35%, *p*-value = 0.009). Between 2021 and 2023, the hospital made satisfactory improvements in leadership and incident management system [from 57% in 2021 to 86% in 2023 (change = 29%, 95% CI: 17–41%, *p* < 0.001)]; risk communication and community engagement [38–88% (change = 50%, 95% CI: 39–61%, *p* < 0.001)]; patient management [63–88% (change = 25%, 95% CI: 14–36%, *p* < 0.001)]; and rapid identification and diagnosis [67–83% (change = 16%, 95% CI: 4.2–28%, *p* = 0.009)]. The hospital made fair but significant improvements in terms of coordination and communication [42–75% (change = 33%, 95% CI: 20–46%, *p* < 0.001)], human resources capacity [33–75% (change = 42%, 95% CI: 29–55%, *p* < 0.001)], continuation of critical support services [50–75% (PD = 25%, 95% CI: 12–38%, *p* < 0.001)], and IPC [38–63% (change = 25%, 12–38%, *p* < 0.001)]. However, there was no or unsatisfactory improvement in terms of surveillance and information management; administration, finance, and business continuity; surge capacity; and occupational and mental health psychosocial support.

**Conclusion:**

Substantial gaps still remain in the hospital's readiness to respond to the COVID-19 outbreak. The study highlights the urgent need for investment in resilient strategies to boost readiness to respond to future outbreaks at the hospital.

## Introduction

Over the past decades, there has been a series of pandemics around the world as a result of various emerging and re-emerging infectious diseases (1, 2). These outbreaks have had dire consequences characterized by unprecedented socioeconomic disruption coupled with strained healthcare systems, especially in resource-poor settings (3, 4). For example, COVID-19 has resulted in an exacerbation of economic challenges due to restricted movement, closure of businesses, increased transaction costs, and a decline in demand for services requiring physical human interaction (5). It also worsened the plight of already vulnerable individuals. For example, among women refugees in Iran and Afghanistan, COVID-19 resulted in increased cases of psychological stress, unwanted pregnancies, depression, suicidal ideation, aggression, disappointment stigma, and rejection (6–9). Mutambara et al. also reported increased domestic violence in South Africa among women refugees (10).

The World Health Organization's (WHO) Africa region has experienced a high burden of infectious diseases. In 2018 alone, there were 96 new disease outbreaks reported to the WHO (11). Between 1976 and 2022, the region experienced 49 Ebola Virus Disease (EVD) outbreaks (12, 13). The WHO also reported a 400% increase in the number of cases of measles in 2022 compared to 2021, and more countries in the region have reported outbreaks of polio and yellow fever (14). As such, the WHO has developed and implemented a series of health system interventions to enable health systems in poor countries to become more prepared to detect and respond to epidemics and prevent shocks to routine health services, an example being the COVID-19 Strategic Preparedness and Response Plan (15). However, we do not know the extent to which countries have implemented the WHO recommendations and, thus, their effectiveness.

Liberia was one of the three heavily affected West African countries during the 2013–2016 EVD outbreak, with 28,000 cases and about 11,000 deaths (16–20). This pandemic exposed the weaknesses in the health system and the unpreparedness of the response to a pandemic of such deadliness in Liberia (21). One of the main causes of the nation's preexisting vulnerabilities was the 14 years of civil unrest that led to damaged infrastructure and equipment, as well as the loss of resources and medical personnel (20–22). However, the country managed to contain the outbreak using a variety of public health interventions, including the development of the country's 2015–2021 Investment Plan for Building a Resilient Health System (23), with the assistance of international partners. Some of these preparedness interventions led to strengthened public health systems (23, 24). However, despite this robustness and improved readiness capacities in public health institutions, health facility-level preparedness in the country is still believed to be inadequate, but no data exist.

The COVID-19 epidemic reached Liberia in 2020 (25), ~6 years after EVD devastated the country's health system and created the first opportunity to test health facility readiness capacities to respond to a large-scale epidemic while retaining routine health services. Despite the national response to the epidemic, COVID-19 rapidly spread across the country and is believed to have affected a large proportion of the population, but the exact extent is unknown due to low testing capacity and mild symptoms in the majority of cases (26). It was also noted that many cases occurred in health facilities despite reports of screening and IPC measures (26). Investigating health facility readiness forms the foundation for instituting interventions to boost preparedness (26), but none have been done in Liberia despite the existence of validated tools.

To understand health facility readiness capacity to respond to epidemics, we assessed the John F. Kennedy Medical Center's (JFKMC) readiness to respond to the COVID-19 pandemic. The JFKMC is Liberia's only tertiary and teaching hospital, established in 1971 (27), and serves an estimated population of just over 5 million people (28). This should help hospital management and policymakers generate contextual solutions that will make significant population-level gains with improved hospital pandemic responses in the future.

## Materials and methods

### Study design

We used the pretest-posttest design in June 2021 and May 2023 to assess improvements to the JFKMC's COVID-19 pandemic response readiness. After the first survey in June 2021, lessons learned were communicated across the facility, followed by the implementation of necessary interventions to address the gaps identified during the survey. We then conducted a second survey in May 2023 using the same survey tools to assess changes in the facility's readiness to respond to the COVID-19 pandemic. The surveys were approved by the Office of the Chief Medical Officer.

### Study site

The JFKMC is Liberia's premier tertiary, referral, and teaching hospital, situated in the capital, Monrovia. It has a capacity of 500 beds but currently has more than 400 functional beds. It was commissioned in 1971 (27). The hospital provides all levels of health care, that is, primary, secondary, and tertiary medical services. The first two services are provided to the communities around it and tertiary health services to the whole of Liberia. It receives referrals from all 15 counties of Liberia, which in 2022 had an estimated population of 5.3 million (28). Funding for the hospital's operations comes mainly from government allotments and fees-for-service, with additional support from both local and international organizations.

### Data collection

We used the WHO COVID-19 Rapid hospital readiness checklist, WHO reference number: WHO/2019-nCoV/hospital_readiness_checklist/2020.2 and license number CC BY-NC-SA 3.0 IGA (29), to assess and guide the hospital's response to the pandemic, first during the peak of the pandemic in June 2021 and then again in May 2023. Additionally, we collected data on the hospital's characteristics, including the presence of an isolation unit and its capacity, the presence and capacity of the intensive care unit, the number of ventilators and other equipment, and the staff dedicated to COVID-19 management. The checklist has 12 thematic areas or components, for a total of 79 indicators. The 12 components are leadership and incident management system; coordination and communication; surveillance and information management; risk communication and community engagement; administration, finance, and business continuity; human resources; surge capacity; continuity of essential support services; patient management; occupational health, mental health, and psychosocial support; rapid identification and diagnosis; and infection prevention and control. It was designed to help policymakers and administrators identify major areas in hospitals that require investment and action, can be used periodically to assess hospitals' capacity to handle pandemics, and can be adapted for use in long-term care facilities (29). It can be used from before the start of an emergency and throughout its various stages (29). Soft copies of the questionnaire and checklist were completed by the authors in consultation with the Epi-surveillance and Infection Prevention and Control (IPC) teams over a period of 2 weeks each time, with additional data being collected from the hospital records. The responses to each of the indicators in the WHO COVID-19 Rapid hospital readiness checklist were classified as not available, partially functional, and fully functional. Non-available was defined as a component that was planned but has not started or does not exist. Partially functional was defined as a component that exists but is not comprehensive enough to achieve all of the core elements required to act. Fully functional was defined as a component that is effectively and efficiently operational.

### Statistical analysis

We report the characteristics of the hospital, followed by a cross-sectional analysis of the level of readiness for COVID-19 management in June 2021 and May 2023. We scored the responses to each of the 79 hospital readiness indicators (fully functional = 1, partially functional = 0.5, and not available = 0) and converted the scores to percentages. We then calculated the score for each of the 12 thematic components as the average of the thematic indicator scores for that thematic component and performed the chi-square test to calculate the change in readiness as a difference (95% CI, *p*-value) between the 2021 and 2023 scores (%s). We performed the same operation to calculate the overall hospital readiness score (%) at each time point and the change (95%CI, p-value) in readiness between 2021 and 2023. The overall hospital readiness scores at each time point were classified as unsatisfactory (< 50%), fair (50–79%), or satisfactory (≥80%), according to the WHO scoring classification. The analysis was performed with Stata version 17.0 (StataCorp, Texas, United States).

## Results

### Hospital characteristics

Table 1 provides a general overview of the hospital and also highlights some specific features that were pertinent to providing holistic medical care to COVID-19 patients but not directly addressed in the WHO COVID-19 Rapid hospital readiness checklist. Of note is that there were no changes in the hospital's bed capacity, with the only notable changes being in the number of staff dedicated to COVID-19 care and IPC, though the overall number of staff in the hospital remained largely unchanged.

**Table 1:** General overview of the John F. Kennedy Medical Center, Monrovia, Liberia.

**Characteristics**	**June 2021**	**May 2023**
Routine bed capacity	410	410
Hospital surge bed capacity	0	0
**Routine bed capacity by department**		
Medical emergency	12	12
Pediatric emergency	16	16
Trauma/accident	14	14
Medicine	46	46
Pediatrics	42	42
Obstetrics/gynecology	160	160
Surgery^a^	50	50
Psychiatry	70	70
**ICU**^b^ **capacity**		
Beds	8	8
Ventilators	2	3
Oxygen concentrators	3	3
Oxygen cylinders	4	5
Clinical staff	15	17
**Clinical staff dedicated to patient care**		
Nursing/midwifery	240	254
Internal medicine	6	6
Infectious disease	1	2
Emergency medicine	1	1
Surgery	11	11
Laboratory	2	3
Imaging/radiology	2	2
COVID-19 case management	2	3
Infection prevention and control	2	11
Public health/epidemiology	0	0
**COVID-19 isolation capacity**		
ICU beds	0	0
Non-ICU beds	1	1
Holding area stretchers	12	12
Ventilators	0	0
Oxygen concentrators	5	8
Oxygen cylinders	4	12
Trained clinical staff dedicated to COVID-19	3	18
Trained IPC staff	3	14
Public health staff	0	0

^a^The hospital has 13 functional operating theaters, hence the rapid surgical bed turnover rate.

^b^Intensive care unit.

### Rapid hospital readiness checklist

#### Hospital readiness scores

The overall hospital readiness for COVID-19 response was fair (mean score in 2021 = 49%, 95% CI: 39–57% and in 2023= 6 9%, 95% CI: 56–81%). The mean change in hospital readiness was 20% (95% CI: 5.7–35%, *p*-value = 0.0087). Between 2021 and 2023, the hospital made satisfactory improvements in leadership and incident management system [from 57% in 2021 to 86% in 2023 (change = 29%, 95% CI:17–41%, *p* < 0.001)]; risk communication and community engagement [38–88% (change = 50%, 95% CI: 39–61%, *p* < 0.001)]; patient management [63–88% (change = 25%, 95% CI: 14–36%, *p* < 0.001)]; and rapid identification and diagnosis [67–83% (change = 16%, 95% CI: 4.2–28%, *p* = 0.0090)]. The hospital made fair but significant improvements in terms of coordination and communication [42–75% (change = 33%, 95% CI: 20–46%, *p* < 0.001)], human resources capacity [33–75% (change = 42%, 95% CI: 29–55%, *p* < 0.001)], continuity of essential support services [50–75% (change = 25%, 95% CI: 12–38%, *p* < 0.001)], and IPC [38–63% (change = 25%, 12–38%, *p* < 0.001)]. However, there was no or unsatisfactory improvement in terms of surveillance and information management [75–75% (change = 0%, 95% CI: −12 to 12%, *p* = 1.000)]; administration, finance, and business continuity [44–44% (change = 0%, 95% CI: −14 to 14%, *p* = 1.000)]; surge capacity [30–30% (change = 0%, 95% CI: −13 to 13%, *p* = 1.000)]; and occupational and mental health psychosocial support [40–40% (change = 0%, 95% CI: −14 to 14%, *p* = 1.000)] (Table 2).

**Table 2:** JFK Hospital readiness scores for COVID-19 response by WHO thematic component in 2021 and 2023.

**Component**	**June 2021**	**May 2023**	Change^*^(95% CI)	***p*-value**
1. Leadership and incident management system	57%	86%	29% (21–49%)	< 0.001
2. Coordination and communication	42%	75%	33% (21–47%)	< 0.001
3. Surveillance and information management	75%	75%	0% (−16 to 16%)	=1,000
4. Risk communication and community engagement	38%	88%	50% (42–65%)	< 0.001
5. Administration, finance, and business continuity	44%	44%	0% (−14 to 14%)	1,000
6. Human resources	33%	75%	42% (30–55%)	< 0.001
7. Surge capacity	30%	30%	0% (−15 to 15%)	1,000
8. Continuity of essential support services	50%	75%	25% (13–40%)	< 0.001
9. Patient management	63%	88%	25% (19–48%)	< 0.001
10. Occupational, mental health, and psychosocial support	40%	40%	0% (−14 to 14%)	1,000
11. Rapid identification and diagnosis	67%	83%	16% (6.0–37%)	0.001
12. Infection prevention and control	38%	63%	25% (12–38%)	< 0.001
Mean score (95%CI)	**49%**	**69%**	**20% (5.7–35%)**	**0.004**

^*^Difference or change in readiness with chi-square test *p*-values.

#### Leadership and incident management systems

During the 2023 survey, the hospital attained satisfactory improvements in leadership from 57% in 2021 to 86% in 2023 (change = 29%, 95% CI: 17–41%, *p* < 0.001) (Table 2). An emergency response plan was developed soon after the first case of COVID-19 in Liberia was announced, and committees were established to lead the hospital's response to the pandemic. The team had experiential advantages that enabled it to rapidly establish and skill up its operations, given its past participation in the 2014–2016 EVD epidemic in Liberia. They identified an emergency operation area with limited resources and established an incidence management system (IMS) that met periodically and participated in the national IMS at the MOH. Another advantage was that the national IMS selected the hospital's Chief Executive Officer (CEO) to serve as the thematic lead of COVID-19 case management, thereby strengthening coordination and communication between the hospital and the national response. A business continuity plan was developed during the pandemic, but no simulation exercises were carried out. Procedures were established for risk management, including the restructuring of clinical services, daily monitoring, risk assessment, and procedures to investigate risks and breaches in the hospital's health security measures. Policies were instituted to ensure daily screening of staff, visitors, and patients and the movement of patients, supplies, and staff within and without the facility (Supplementary Table 1A). From the beginning, measures were put in place to have patients suspected of having COVID-19 transferred to treatment centers.

#### Coordination and communication

In the area of coordination and communication, the hospital scored fairly 42% in 2021 and 75% in 2023 (change = 33%, 95% CI: 20–46%, *p* < 0.001) (Table 2). Following the initial assessment, standard operating procedures were developed, and a focal person was identified. A directory was developed, and the team set up to lead the hospital's response to the pandemic was tasked with keeping the staff and administration updated (Supplementary Table 1A). The team, however, lacked resources for its activities, and team members were using their cellphones for this purpose while the hospital provided data at times. Several training sessions and update meetings were held with staff, and approximately 90% of the hospital's staff, both clinical and non-clinical, were briefed and trained on COVID-19 emergency policies and procedures. The head of the team, together with several other members, was trained by the MoH and County Health Team (CHT) and served as the spokesperson of the team. However, the hospital did not have regular updates for the public, despite having a weekly radio talk show hosted by the national broadcaster, the Liberian Broadcasting System, as this was the responsibility of the MoH. However, at the facility level, designated staff provided health promotional messages and mandatory COVID-19 response measures every morning to patients and visitors in the Out Patient Department (OPD), at the facility's main entry point, visitor-waiting areas, and COVID-19 screening points. Coordination was maintained with key stakeholders, such as suppliers of oxygen and pharmaceuticals.

#### Surveillance and information management

The hospital's fair (75%) performance in surveillance and information management remained unchanged (change = 0%, 95% CI: −12 to 12%, *p* = 1.000) (Table 2). The already existing Epi-Surveillance team was modified to lead the hospital's response to the pandemic with the addition of other key staff with representations from nearly all the hospital units. The team was responsible for case identification, investigation, reporting, and data storage. This was achieved through regular COVID-19 screening on standard COVID-19 triage forms at the JFK triage. Within the hospital, the clinicians specifically screened all patients, regardless of reason for clinic visit or admission, for COVID-19 and reviewed and included the COVID-19 assessment reports in the patient charts. Standardized forms and standard operating procedures, provided by the CHT, were used for information gathering and dissemination. They also created awareness through the printing of COVID-19 case definitions that were posted on every unit, and together with the administration, they modified the triage forms to include COVID-19-specific information (Supplementary Table 1B). Additionally, the team generated contact line listings specifically for staff, which were sent to the IPC team for risk assessment. However, there were no formal channels developed for collecting feedback from patients and visitors. The hospital's administration, the CHT, and the MoH received data gathered by the Epi-surveillance team on individuals who tested positive and their contacts, which was then compiled and shared with other stakeholders and the public.

#### Risk communication and community engagement

The hospital made substantial and satisfactory improvements in risk communication and community engagement, which improved from 38% in 2021 to 88% in 2023 (change = 50%, 95% CI: 39–61%, *p* < 0.001) (Table 2). Most of the risk communication protocols received for the MoH had messages directed toward healthcare workers, while those with messages meant for patients, their visitors, and the general public were limited. Key messages were modified as needed as new information was being released, mainly by the WHO, and these new messages were shared with the concerned staff (Supplementary Table 1B).

#### Administration, finance, and continuity

No changes were noted in the overall scores of this thematic area [44–44% (change = 0%, 95% CI: −14 to 14%, *p* = 1.000)] (Table 2; Supplementary Table 1B). There were no legal procedures or financial mechanisms specifically created for COVID-19, even though special interventions were made to address some challenges, such as reducing procurement turnaround times. Additionally, there was no liability or insurance coverage for staff managing COVID-19 suspects and confirmed cases. The hospital, however, facilitated the care of exposed staff and those who got infected, including providing basic supplies and food when they were admitted into the COVID-19 unit. No staff turnover surge plan was in place, and as such, absenteeism due to COVID-19 infection or exposure created gaps that affected services. No funding was available for the deployment of emergency staff. Attempts were made to reassign staff to various areas or units to manage cases of COVID-19 while continuing to provide routine healthcare services. There was no formal system of billing COVID-19 suspects or confirmed cases, though most had part of their bills eventually waived. Stable patients being followed up in the outpatient department were given prescriptions lasting several months and advised to call their primary doctors before returning to the hospital unless it was an emergency.

#### Human resources

Fair improvements [from 33% in 2021 to 75% in 2023 (change = 42%, 95% CI: 29–55%, *p* < 0.001)] were made to human resources management between 2021 and 2023 (Table 2; Supplementary Table 1C), largely due to staffing-needs evaluations being conducted, resulting in only a few disruptions to the routine services provided by the hospital. Staff were sometimes repurposed and assigned to problem areas as needed. Staff at risk of severe COVID-19, for example, the older adult, diabetics, and pregnant women, were given forced leave of absence. All staff were also trained on COVID-19-specific IPC techniques, and attempts were made to ensure staff safety, though this was hampered by the lack of appropriate personal protective equipment (PPE).

#### Surge capacity

The hospital had surge and replenishment plans, but due to increased demand and limited supplies, the traditional sources of supplies, especially PPEs, were no longer reliable, thus the overall score for this thematic area was poor [30–30% (change = 0%, 95% CI: −13–13%, *p* = 1.000)] (Table 2; Supplementary Table 1C). Most of the PPEs used at the hospital at the beginning of the pandemic were supplies remaining from stocks acquired during the Ebola outbreak of 2013–2016. Equipment, especially oxygen concentrators, were mostly acquired through donations. Some organizations donated oxygen tanks and offered to procure oxygen at regular intervals. However, there were no short-term plans to create bed space for suspected or confirmed COVID-19 cases. The cases were only to be stabilized while awaiting transfer to the designated COVID-19 treatment centers.

#### Continuity of essential support services

The management of the hospital made attempts to ensure the continuity of routine and essential services, with the readiness score increasing from 50% in 2021 to 75% in 2023 (change = 25%, 95% CI: 12–38%, *p* < 0.001) (Table 2; Supplementary Table 1C) in 2021 and 2023, respectively. Contingency plans were put in place to ensure fewer stock-outs of essential drugs, medical supplies, and other essential supplies such as detergents and food. The disruption of global supply chains, however, made it difficult to have certain items always in stock, such as face shields, face masks, gowns, and plastic aprons.

#### Patient management

The hospital scored 63% in the initial assessment and then 88% in 2023 (change = 25%, 95% CI: 14–36%, *p* < 0.001) (Table 2; Supplementary Table 1C). Initially, the use of symptomatic screening tools for COVID-19 had limited effectiveness because a substantial number of COVID-19 patients are asymptomatic or have mild disease. As a result, some cases were missed and admitted to the hospital, while additional cases emerged from community or nosocomial exposure. Point-of-care testing was later added to screening at points of entry, thereby substantially improving patient management and boosting staff confidence.

The hospital was not designated as a treatment center; the initial plan was to receive, diagnose, and transfer cases of COVID-19. This, however, changed when the designated treatment center was overwhelmed and the hospital had nowhere to transfer patients. As a result, a total of 69 patients were admitted and treated for COVID-19 using updated protocols in the medical ER over a period of 6 weeks, starting during the third week of June 2021. Later, the management of the hospital began the construction of an eight-bed isolation unit next to the emergency room (ER) to provide a more suitable space to hold and begin treatment, not only for COVID-19 patients but also for other communicable diseases of concern such as Lassa fever and measles. This structure, whose construction began ~2 years ago, is yet to be completed and utilized. The major challenge cited for the delays has been a lack of funding. It is also because of a lack of funding that the hospital has been unable to construct a standalone isolation unit.

#### Occupational health, mental health, and psycho-social support

The hospital scored 40% in both 2021 and 2023 (change = 0%, 95% CI: −14–14%, *p* = 1.000) (Table 2; Supplementary Table 1D). Although the staff were well-trained, they were not well-equipped and protected to provide initial medical screening and care to people with suspected, probable, or confirmed COVID-19 (Supplementary Table 1D). There were no ICU beds or ventilators for the management of severe COVID-19, no surge capacity or public health staff (Table 1), frequent stockouts of PPE, NIOSH-approved respirators, other essential COVID-19 supplies, and a lack of pulse oximeters at screening points. Staff who became infected, injured, or developed severe COVID-19 had no insurance coverage (Supplementary Tables 1D, E). All infected staff were encouraged to self-isolate or be referred to the national COVID-19 treatment unit, and some support was provided by the hospital. This included daily feeding and refreshments for those admitted to the national treatment center and basic groceries for the families in isolation. Team leads and unit heads were key in keeping the morale of the staff high. They tried to ensure that the patients were catered to by staff with limited resources without overly endangering them. The Social Services Department, a department made up largely of staff with psycho-social counseling skills, was often called upon to counsel exposed staff who had been quarantined. Because of their small numbers, the counseling was limited to affected staff. Counseling of patients and relatives was done mostly by the physicians and nursing staff. However, no formal mental health and psychosocial service protocols were available.

#### Rapid identification and diagnosis

Satisfactory improvement was made between the 2021 and 2023 surveys [67–83% (change = 16%, 95% CI: 4.2–28%, *p* = 0.009)] (Table 2; Supplementary Table 1D) regarding rapid identification and diagnosis of COVID-19 cases. Staff on the Epi-Surveillance team were adequately trained by the MoH and CHT on how to rapidly diagnose COVID-19 using WHO guidelines, and these were updated as needed and reported formally to the CHT. Heads of various clinical units were also drafted into the response team, and they had effective means of communication among them. Patients were triaged at multiple points in the hospital, beginning at the main gate, then at the point of registration and areas where vital signs were checked. The Epi-Surveillance team, together with laboratory technicians, was trained in standardized procedures for collecting samples and transferring them to the reference laboratory, according to current recommendations. At the time of the first assessment, the hospital could not carry out any COVID-19 tests, including rapid tests. Samples for COVID-19 polymerase chain reaction (PCR) testing were taken to the national reference laboratory, with results only becoming available 2–3 days later. As the number of cases increased during the epidemic, timely case identification became a challenge, as the surveillance team became overwhelmed as they still had to continue carrying out regular surveillance of priority diseases, mostly Lassa fever, measles, and cholera.

After the 2021 survey, the hospital established rapid point-of-care PCR testing for COVID-19 testing in collaboration with the US National Institute of Health-funded clinical-research Partnership for Research on Vaccines and Infectious Diseases in Liberia (PREVAIL). Test results were available in less than a day for clinical decision-making. However, the COVID-19 testing was unavailable during evening and night hours, weekends, and holidays. The hospital lacked the capacity to conduct PCR testing; hence, the testing gaps during after-hours, weekends, and holidays were never addressed.

#### Infection prevention and control

The hospital's overall IPC score was poor [from 38% in 2021 to 63% in 2023 (change = 25%, 12–38%, *p* < 0.001)] (Table 2). The IPC team implemented IPC protocols from the MoH and CHT and other health security precautions (Supplementary Table 1E). Health services were restructured to enhance health security. The facility ensured that everyone accessing it entered through one point, and mandatory hand-wishing and mask mandates were implemented. COVID-19 symptom screening and later on, rapid point-of-care testing were established close to the entrance of the hospital to screen all patients, visitors, and hospital staff. However, several lapses were identified (Supplementary Table 1E). On several occasions, improvised hand-washing stations were either without soap or water, and hand sanitizers were not always available. Face masks were in short supply, and though everyone was forced to produce and wear one at the hospital's main gate, once inside the hospital compound, most people were seen without them. Exits from the hospital also occurred at one exit gate. Within the hospital, patient beds were placed 3 m apart according to the recommendation, but appropriate PPE for each procedure was not always available. Additionally, staff were encouraged to call their supervisors if they developed any symptoms suggestive of COVID-19 so they could be allowed to go for testing at centers nearest to them without presenting to the hospital. An open space outside the medical emergency room was designated for suspected and confirmed cases while awaiting transfer to a treatment unit. In the maternity center, an unused operating room near the emergency room was kept for suspected and confirmed cases in need of treatment. Protocols were available for the movement of patients within the hospital and transport by ambulance. Those working in direct contact with patients did not have their names recorded formally. Protocols on how to manage the bodies of those who died of COVID-19 were available from the beginning and enforced.

IPC protocols for waste management, including labeled/standard colored bins, waste sorting, incineration, and sharp boxes per bed were inconsistently followed. Although mask mandates and hand hygiene were instituted, the protocols were not followed consistently; water and soap for hand washing were not available in all key locations; some patients, staff, and visitors were observed without masks during the surveys; and use of a new pair of gloves and hand washing was not performed in between patients at all times due to inadequate supplies.

## Discussion

The hospital's overall readiness in 2023 was fair (69%), resulting from inadequate improvements (20%) made to address gaps identified during the first survey in 2021 (49%). Despite the existence of assessment tools, our study is among the few to assess health facility readiness for COVID-19 response in sub-Saharan Africa and the first in Liberia.

Emergency preparedness, a state of being in constant readiness, has four phases, namely, the mitigation, preparation, response, and recovery phases (30). An emergency response plan is expected to result in a robust and efficient response to public health threats with no, or minimal, disruptions to routine services. After 1 year of the pandemic, the WHO reported that about 90% of countries worldwide still had substantial disruptions to the provision of essential health services as a result of inadequate emergency preparedness (31).

Our findings revealed that, though there was a significant improvement in the overall readiness score of the hospital over the 2 years, these improvements were non-uniform, with significant gaps remaining. Significant improvements were noted in the hospital's leadership and incident management systems; risk communication and community engagement; patient management; and rapid identification and diagnosis. These achievements could be attributed to the fact that the hospital already had an active Epi-Surveillance team that was actively looking out for cases of Lassa fever, measles, and cholera, as there were outbreaks of these diseases in Liberia at the time. The team therefore simply needed slight modifications and training on COVID-19-related responses. Being the only hospital in the country with consultant physicians, including infectious disease consultants, the sourcing and sharing of up-to-date guidelines on COVID-19 was relatively easy. The COVID-19 pandemic has been described as a catalyst for change, ushering in accelerated changes and the adoption of specific public health interventions (32). It is thus anticipated that future pandemics will be met with robust, effective, and efficient responses. Improvements in rapid identification and diagnosis helped improve decisions for care as the time to receive results was reduced from 3 days to a few hours. The challenges of testing for COVID-19 at the beginning of the pandemic were not unique to the hospital; many African countries faced the same challenges, which may have resulted in under-reporting of cases and unnecessary quarantine (33).

Significant gaps remained for some components, with unsatisfactory end-of-review scores noted in administration, finance, and business continuity; surge capacity; and occupational health, mental health, and psychosocial support. These gaps generally highlight the pre-existing gaps the hospital had prior to the pandemic.

Our findings are not unique to the JFKMC and Liberia, as similar observations have been made in hospitals across the globe. A survey of 20 hospitals in Nigeria for 5–8 months after the country reported its index case showed that most hospitals were not adequately prepared to respond to the COVID-19 pandemic, though the overall readiness scores were higher than those observed at the JFKMC. Most hospitals in that survey had gaps in infrastructure, equipment, human resources, processes, and procedures related to the COVID-19 response (34). Even developed countries were unprepared for the impact of the pandemic. In the United States of America, for example, the Department of Health and Human Services did a national pulse survey in March 2020 and reported that most of the challenges faced by hospitals were centered on testing and caring for patients with known or suspected COVID-19 while keeping healthcare workers safe. There were also significant challenges in maintaining or expanding hospitals' capacity to treat patients with COVID-19. Hospitals had to request assistance in acquiring personal PPEs, testing, staffing, equipment and supplies, and funding from the department (35). It was reported that supply chain disruptions were resulting in as much as 6-month delays in the delivery of much-needed medical supplies (35). Limited funding appears to be a common factor in the limited responses to the pandemic globally. In 2021, the WHO reported that 43% of countries globally cited financial challenges as major causes of disruptions in service utilization, with developed countries not being spared (31).

A facility of this size and a tertiary teaching hospital are expected to at least have a large enough isolation unit or infectious disease hospital that is adequately manned and equipped (36). An isolation unit is key to interrupting the transmission of communicable diseases (37). Currently, no such facility is available in the hospital. Instead, an eight-bed holding unit is presently under construction but will not be able to serve as a treatment center for any of the communicable diseases with epidemic potential due to its size and the lack of a supporting laboratory and other necessary amenities. It appears that having adequate space for isolation is a major challenge in some African countries. Uganda, for example, was forced to convert its major psychiatric hospital into an isolation unit as constructing an isolation unit was deemed not feasible due to funding limitations (38). High-income countries could easily and rapidly construct new facilities, as was witnessed in China (39). However, in the City of New York, administrators found more success in repurposing existing hospital space than building field hospitals, as these hospitals would have required replicating staffing and equipment needs (40). During the period reviewed, no space was available to accommodate a surge in cases, and some services had to be reduced or suspended to admit COVID-19 cases, including elective surgical cases. A review of the hospital's admission records over the past 5 years (unpublished data) shows that there has been a steady increase in the bed occupancy rate, with the current rates close to 100%. This limited the flexibility and ability of the hospital to adapt to COVID-19-related needs without disrupting routine services. Though the facility has an ICU with three ventilators, these were never used for COVID-19 cases, as this would have meant no other critical cases could be managed in the ICU. In a survey of 13 hospitals in Malawi in early 2020, it was revealed that several hospitals could provide both non-invasive ventilation and mechanical ventilation to COVID-19 patients (41), yet the JFKMC reserved its ventilators for non-COVID-19 patients.

Ensuring adequate stocks of PPEs was a huge undertaking, as there was a huge surge in the demand for and utilization of gloves, facemasks, and goggles. The usual vendors and suppliers of these materials had limited stocks and ran out of these items often. This led to staff improvising at times, for example, reusing face masks or washable cloth face masks and using non-medical plastic gloves. Increased global demand for PPEs and global supply chain disruptions are largely to blame for these shortages (42, 43).

The need for psychosocial support for medical staff cannot be overemphasized, as has been witnessed in other African countries (44). As a direct consequence of the COVID-19 pandemic, witnessing the suffering patients went through and the fear of being infected made the staff at the hospital vulnerable to psychological stress, and the Social Services department was tasked with counseling and providing psychosocial services to affected staff. This worked well in the beginning; however, as the number of affected staff increased, the department became overwhelmed and could not offer its services to all those in need. Irandoost et al., after reviewing the challenges and adaptation strategies of nurses caring for COVID-19, concluded that more attention should be paid to nurses' physical and mental wellbeing, among other things (9).

Administratively, the JFKMC has three pillars, namely, clinical services, teaching, and research (45). The WHO states that to manage cases of COVID-19 adequately, a facility needs to have sufficient oxygen, ICU beds, ventilators, isolation space, and PPE, among other resources (46). A closer look at this list shows that these are basic things that a tertiary hospital should have for it to meet its clinical service, teaching, and research objectives. The lack of a research laboratory, an infectious disease unit, and limited equipment such as ventilators certainly affect all three pillars negatively. This is more worrisome considering that the facility is still recovering from the worst African EVD outbreak of 2014 (16, 18) and the only positive and significant change to the hospital is its triaging system and IPC practices, which again are not perfect.

Some limitations of this review are worth noting. The data was collected by the authors in consultation with other relevant persons employed by the hospital, which could have introduced some bias. Second, the first review, which served as the baseline review, was conducted 1 year after the index case was seen at the hospital. The review, therefore, did not account for the changes made in response to the pandemic before the review. Additionally, this study was limited to one facility only, yet the national response to COVID-19 involved other facilities. As such, our findings may not be generalized to other facilities in the country.

Nonetheless, this study is the first of its kind at the JFKMC, and it formally highlights the hospital's strengths while also bringing to light the glaring challenges that the hospital needs to address if it is to successfully meet its mission and goals as well as fend off the impact of future epidemics. Assessing the hospital at two time points provides insight into how much investment is needed for each thematic area. Having experienced the worst Ebola outbreak in history and now the COVID-19 outbreak, it is expected that lessons drawn from these experiences should be used to build a robust, adaptive, and resilient tertiary hospital.

## Conclusion

This study provides a unique understanding of the strengths and weaknesses of the hospital regarding COVID-19 readiness. It highlights the urgent need for cost-effective, innovative, all-encompassing strategies to respond to future outbreaks at the hospital, with a special focus on its administration, finance, and business continuity; surge capacity; and occupational health, mental health, and psychosocial support. Additionally, in the long term, health system strengthening is needed for the provision of improved clinical services, teaching, and research at the JFKMC to ensure an improved capacity to prepare, mitigate, respond, and recover from future shocks.

Further studies would need to be carried out to identify factors associated with the successes and failures to implement the components included in the WHO COVID-19 Rapid hospital readiness checklist so that specific interventions can be devised to address the hospital's weaknesses while further bolstering its strengths.

## Data availability statement

The raw data supporting the conclusions of this article will be made available by the authors, without undue reservation.

## Ethics statement

Ethical review and approval was not required for the study on human participants in accordance with the local legislation and institutional requirements. Written informed consent from the participants was not required to participate in this study in accordance with the national legislation and the institutional requirements.

## Author contributions

IW: Conceptualization, Data curation, Formal analysis, Investigation, Methodology, Project administration, Resources, Supervision, Validation, Visualization, Writing—original draft. SC: Writing—review & editing. TK-T: Data curation, Formal analysis, Writing—review & editing. FG: Data curation, Writing—review & editing. YB-K: Data curation, Writing—review & editing. JB-G: Conceptualization, Data curation, Formal analysis, Investigation, Methodology, Project administration, Visualization, Writing—original draft. JM: Formal analysis, Writing—original draft, Writing—review & editing.
