# Supplementary material for: A review of the John F. Kennedy Medical Center's response to the COVID-19 pandemic in Liberia
Source: Front Public Health. 2024 Jan 9;11:1258938. doi: 10.3389/fpubh.2023.1258938 (PMC10803570; doi:10.3389/fpubh.2023.1258938)
Supplement: Supplementary file 1 [file Table_1.docx]

**Supplementary Tables**

**Table 1A: JFK Medical Center readiness scores for COVID-19 response leadership and coordination indicators in 2021 and 2023**

| **INDICATOR**^1^ | **2021** | **2023** |
| --- | --- | --- |
| **1. Leadership and incident management system** | **%** | **%** |
| 1.1 Hospital has a well-established emergency response plan for COVID-19 | 7.1 | 14.3 |
| 1.2 Hospital has a physical area for a hospital emergency operations centre | 7.1 | 7.1 |
| 1.3 Hospital has a designated incident manager | 7.1 | 14.3 |
| 1.4 Designated leads are assigned to the COVID-19 incident management system team | 14.3 | 14.3 |
| 1.5 Hospital has a tested business continuity plan for COVID-19. | 0 | 7.1 |
| 1.6 Hospital has mechanisms to coordinate with national and local authorities | 14.3 | 14.3 |
| 1.7 All documents on COVID-19 risk management are available for use by staff. | 7.1 | 14.3 |
| **2. Coordination, internal and external communication** |  |  |
| 2.1 Hospital has a COVID-19 internal communications plan | 0 | 16.7 |
| 2.2 Hospital has communications equipment and systems for addressing the COVID-19 pandemic | 8.3 | 8.3 |
| 2.3 All staff have been briefed and trained on COVID-19 emergency policies and procedures | 8.3 | 8.3 |
| 2.4 Hospital COVID-19 incident management system team has activated mechanisms for coordination and communication with stakeholders | 16.7 | 16.7 |
| 2.5 Hospital has an official hospital spokesperson for COVID-19 information | 8.3 | 16.7 |
| 2.6 Hospital has a list of all stakeholders involved in COVID-19 management | 0 | 8.3 |

1 WHO Rapid hospital readiness checklist for COVID-19

**Table 1B: JFK Hospital readiness scores for COVID-19 response surveillance, risk communication, and administration indicators in 2021 and 2023**

| **INDICATOR**^1^ | **2021** | **2023** |
| --- | --- | --- |
| **3. Surveillance and hospital information management** | **%** | **%** |
| 3.1 Staff have been trained in definitions of COVID-19 cases, close contacts and the quarantine system. | 16.7 | 16.7 |
| 3.2 Standardized COVID-19 case forms are available | 16.7 | 16.7 |
| 3.3 Hospital has standard operating procedures addressing the collection, confirmation and validation of COVID-19 data | 16.7 | 16.7 |
| 3.4 Hospital has designated staff to collect, analyse and disseminate COVID-19 related data | 16.7 | 16.7 |
| 3.5 Hospital ensures proper documentation and secure storage of COVID-19 hospital information | 8.3 | 8.3 |
| 3.6 Hospital has mechanism for collecting COVID-19 related feedback from patients and visitors | 0 | 0.0 |
| **4. Risk communication and community engagement** | % | % |
| 4.1 COVID-19 risk communication protocols about IPC^2^, including SOPs^3^, are available for use by all | 12.5 | 12.5 |
| 4.2 Key messages for use in COVID-19 risk communication have been developed and are regularly updated | 12.5 | 25.0 |
| 4.3 Designated staff regularly update risk communication materials and procedures | 0 | 25.0 |
| 4.4 Staff are briefed regularly about COVID-19 risk communication messages and community engagement actions | 12.5 | 25.0 |
| **5. Administration, finance and business continuity** | % | % |
| 5.1 All legal procedures for administration and financial mechanisms are in place for COVID-19 management | 6.3 | 6.3 |
| 5.2 Administrative policies and guidance are available to staff to cope with the COVID-19 pandemic. | 6.3 | 6.3 |
| 5.3 Liability and insurance coverage and procedures for COVID-19 management have been reviewed | 0 | 0.0 |
| 5.4 A system is in place to waive user fees for health care for COVID-19 cases | 6.3 | 6.3 |
| 5.5 Staff turnover and absenteeism have been incorporated into the hospital’s corporate strategy | 0 | 0.0 |
| 5.6 The hospital's incident management system team has ways and means for assessing and identifying increasing COVID-19 caseload. | 6.3 | 6.3 |
| 5.7 A COVID-19 plan is available to potentially refer or outsource care of non-critical patients | 12.5 | 12.5 |
| 5.8 A hospital’s business continuity plan has been developed and tested to address the COVID-19. | 6.3 | 6.3 |

1 WHO Rapid hospital readiness checklist for COVID-19

2 Infection Prevention and control

3 Standard operating procedures

**Table 1C: JFK Hospital readiness scores for COVID-19 response human resources surge capacity, continuity of essential services, and patient management indicators in 2021 and 2023**

| **INDICATOR**^1^ | **2021** | **2023** |
| --- | --- | --- |
| **6. Human resources** | % | % |
| 6.1 The staff directory has been updated | 0.0 | 16.7 |
| 6.2 Staff have been briefed, trained, and take part in exercises relevant to their COVID-19 areas of work | 16.7 | 16.7 |
| 6.3 Hospital has estimated the current human resources capacity to respond to COVID-19 caseload. | 0.0 | 8.3 |
| 6.4 The hospital has identified the optimum number of staff | 0.0 | 8.3 |
| 6.5   Systematic procedures are in place to support repurposing and reassignment of staff | 8.3 | 16.7 |
| 6.6   Optimum procedures are in place to monitor occupational health hazards and ensure the safety of staff | 8.3 | 8.3 |
| **7. Surge capacity** | % | % |
| 7.1 Hospital has surge plan | 10.0 | 10.0 |
| 7.2 The hospital is part of the central surge mechanism or system. | 0.0 | 0.0 |
| 7.3 Procedures are in place to ensure management of the COVID-19 surge supply chain | 10.0 | 10.0 |
| 7.4 The hospital has an arrangement with the Ministry of Health or its equivalent to procure equipment necessary for the surge | 10.0 | 10.0 |
| 7.5 A surge roster of qualified human resources is available and has been updated | 0.0 | 0.0 |
| **8. Continuity of essential support services** | % | % |
| 8.1 Hospital identified and prioritized essential services that should be available at all times | 16.7 | 16.7 |
| 8.2 Hospital has identified backup resources to maintain essential support services | 8.3 | 8.3 |
| 8.3 Hospital inventory, stockpile and maintenance systems are in place | 8.3 | 8.3 |
| 8.4 Hospital has identified potential security challenges and has a mitigation plan for security risks | 8.3 | 8.3 |
| 8.5 The hospital has tested an expansion plan for clinical management | 0.0 | 16.7 |
| 8.6 The hospital information management system is available | 8.3 | 16.7 |
| **9. Patient management** | % | % |
| 9.1 Hospital has an updated protocol for providing essential health care services to patients with COVID-19 | 12.5 | 12.5 |
| 9.2 Procedures are available and functional for receiving and transferring patients within the hospital | 25.0 | 25.0 |
| 9.3 Staff have protocols for treatments that may be administered only in the context of ethically approved protocols | 12.5 | 25.0 |
| 9.4 Staff are implementing IPC^2^ protocols and safe hospital network and transportation services for referral | 12.5 | 25.0 |

1 WHO Rapid hospital readiness checklist for COVID-19

2 Infection prevention and control

**Table 1D: JFK Hospital readiness for COVID-19 response occupational health and rapid identification and diagnosis indicators in 2021 and 2023**

| **INDICATOR**^1^ | **2021** | **2023** |
| --- | --- | --- |
| **10. Occupational health, mental health and psychosocial support** | % | % |
| 10.1 Staff are protected, well trained and equipped to provide initial medical care to suspected, probable or confirmed COVID-19 cases | 10.0 | 10.0 |
| 10.2 Hospital has policies about and capacities to manage occupational safety and health | 10.0 | 10.0 |
| 10.3 Appropriate mental health and psychosocial support services are available for staff, caregivers, their families and patients | 10.0 | 10.0 |
| 10.4 SOPs for mental health screening amongst COVID-19 patients/residents, their families, caregivers, and hospital staff are ready and available | 0.0 | 0.0 |
| 10.5 All staff have been trained in basic occupational safety and health measures and psychological first aid | 10.0 | 10.0 |
| **11. Rapid identification and diagnosis** | % | % |
| 11.1 Staff trained in accurate, rapid identification and timely screening of suspected COVID-19 cases | 16.7 | 16.7 |
| 11.2 A communication and monitoring system is in place that allows for timely alerts and reporting of suspected COVID-19 cases in any area of the hospital | 16.7 | 16.7 |
| 11.3 A triage procedure is in place in the emergency department | 16.7 | 16.7 |
| 11.4 Staff are trained in standardized procedures for collecting and transferring samples | 16.7 | 16.7 |
| 11.5 Hospital has standardized systems for COVID-19 testing, supported by assured access to reagents and test kits. | 0.0 | 8.3 |
| 11.6 Information and posters about PPE^2^ and biosafety measures are posted strategically in the laboratory and reception areas | 0.0 | 8.3 |

1 WHO Rapid hospital readiness checklist for COVID-19

2 Personal protective equipment

**Table 1E: JFK Hospital readiness scores for COVID-19 response infection prevention and control indicators in 2021 and 2023**

| **INDICATOR**^1^ | **2021** | **2023** |
| --- | --- | --- |
| **12. Infection prevention and control** | % | % |
| 12.1 IPC^2^ protocols with standardized procedures for managing COVID-19 are available and functional, and all hospital staff have been trained in the protocols | 3.1 | 3.1 |
| 12.2 Adequate PPE^3^ is available and easily accessible | 3.1 | 3.1 |
| 12.3 Staff are trained to recognize and screen all suspected COVID-19 cases | 3.1 | 6.3 |
| 12.4 Designated isolation areas are available | 3.1 | 3.1 |
| 12.5 Airborne isolation room is available | 0.0 | 0.0 |
| 12.6 Standard and transmission-based precautions are applied | 3.1 | 3.1 |
| 12.7 Staff are using airborne precautions during aerosol-generating procedures | 3.1 | 3.1 |
| 12.8 Appropriate measures   are in place at points of entry and are routinely available in all areas of the hospital | 3.1 | 6.3 |
| 12.9 Posters of good hygiene practices are available in strategic locations around the hospital | 0.0 | 3.1 |
| 12.10 A protocol is available about how to transportation of COVID-19 patients with the hospital | 0.0 | 3.1 |
| 12.11 Staff have been trained on IPC using technical guidance from WHO | 3.1 | 6.3 |
| 12.12 A policy has been implemented that ensures all hospital beds are placed at least 1m apart | 3.1 | 3.1 |
| 12.13 All surfaces in the hospital and in ambulances are routinely cleaned and disinfected | 3.1 | 3.1 |
| 12.14 The hospital has infrastructure and a protocol in place for waste management | 3.1 | 6.3 |
| 12.15 A printed or electronic record of all essential people entering a COVID-19 patient’s room is available and maintained | 0.0 | 3.1 |
| 12.16 Physical space and guidelines are available for managing the bodies of COVID-19 victims | 3.1 | 6.3 |

1 WHO Rapid hospital readiness checklist for COVID-19

2 Infection Prevention and control

3 Personal protective equipment
